# Preparation of Reference Material 8504, Transformer Oil

**DOI:** 10.6028/jres.110.086

**Published:** 2005-12-01

**Authors:** Dianne L. Poster, Michele M. Schantz, Stephen A. Wise

**Affiliations:** National Institute of Standards and Technology, Gaithersburg, MD, 20899-8392

**Keywords:** Aroclors, diluent, diluent oil, PCBs, Reference Materials, transformer oil

## Abstract

A new reference material (RM), RM 8504, has been prepared for use as a diluent oil with Aroclors in transformer oil Standard Reference Materials (SRMs) 3075 to 3080 and SRM 3090 when developing and validating methods for the determination of polychlorinated biphenyls (PCBs) as Aroclors in transformer oil or similar matrices. SRMs 3075-3080 and SRM 3090 consist of individual Aroclors in the same transformer oil that was used to prepare RM 8504. A unit of RM 8504 consists of one bottle containing approximately 100 mL of transformer oil. No additional constituents have been added to the oil.

## 1. Introduction

A new Reference Material (RM) consisting of transformer oil has been developed. RM 8504, Transformer Oil, is intended to be used as a diluent oil with transformer oil Standard Reference Materials (SRMs) 3075 to 3080 and SRM 3090 [1] when developing and validating methods for the determination of polychlorinated biphenyls (PCBs) as Aroclors^1^ in transformer oil or similar matrices. This suite of Aroclor transformer oil SRMs consists of individual Aroclors in the same transformer oil that was used to prepare RM 8504 and is intended for use in the determination of PCBs in oil. These transformer oil SRMs have been developed to replace SRM 1581, PCBs in Oil, [2] which is no longer available. SRM 1581 was intended for calibrating instruments and validating methods used in the determination of PCBs in motor and transformer oils. The PCBs were present as Aroclor 1242 and Aroclor 1260 in both motor and transformer oil at concentrations near 100 µg/g. Two bottles containing approximately 90 mL each of PCB-free diluent motor oil and transformer oil also were part of a unit of SRM 1581. RM 8504 is intended to replace the diluent transformer oil of SRM 1581. The preparation of RM 8504 is described below.

## 2. Materials and Methods

The transformer oil used in the preparation of this RM was obtained from a commercial source. The RM was prepared at NIST by distributing transformer oil (Univolt 60, Exxon) into 100 mL amber glass bottles using an automated dispensing machine. The bottles, once filled with approximately 100 mL oil, were then capped with Teflon-lined screw-caps.

An aliquot of transformer oil from the drum of the Exxon Univolt 60 transformer oil was examined for traces of PCBs as would be evident by capillary gas chromatography with electron capture detection (GC-ECD). The aliquot of oil was first placed on aminopropyl solid phase extraction (SPE) columns, eluted with hexane, concentrated, and analyzed by GC-ECD equipped with a capillary column coated with a non-polar stationary phase [5 % (mole fraction) phenyl methylpolysiloxane, DB-5, J&W Scientific, Folsom, CA].

Additional aliquots from six bottles of RM 8504, selected according to a stratified random sampling scheme, were analyzed by GC-ECD equipped with not only a DB-5 capillary column (described above) but also one with a relatively non-polar stationary phase (DB-XLB, J&W Scientific, Folsom, CA). Prior to gas chromatography, these samples were processed using analytical methods used for the determination of PCBs in transformer oil. Specifically, samples were placed on aminopropyl SPE columns and eluted with hexane. The concentrated eluants were then fractionated by liquid chromatography using a semi-preparative aminopropylsilane column with hexane as the mobile phase. This is the same approach used for the determination of the concentrations of Aroclors in SRMs 3075 to 3080 and SRM 3090 [1]. Two additional aliquots of RM 8504 were processed as above with the amount of the evaporated extracts targeted to be at a level similar to that obtained during typical oil sample analyses (about 0.2 g, exact mass known). After evaporation, these samples were injected into a GC-ECD equipped with a DB-5 column using splitless injection (1 µL) with a split at 0.5 min at a flow rate of 90 mL/min to mimic typical oil sample injections.

Selected Aroclors in transformer oil were analyzed by GC-ECD to determine a detection limit of PCBs, as Aroclors, in the transformer oil. Aliquots from dilutions of selected Aroclors in transformer oil (SRMs 3077 and 3075) with toluene were analyzed directly (no cleanup) by GC-ECD using conditions used for oil analyses [1] and a DB-5 column. These aliquots were injected into a GC-ECD using splitless injection (1 µL) with a split at 0.5 min at a flow rate of 90 mL/min.

## 3. Results and Discussion

**Fig. 1:**
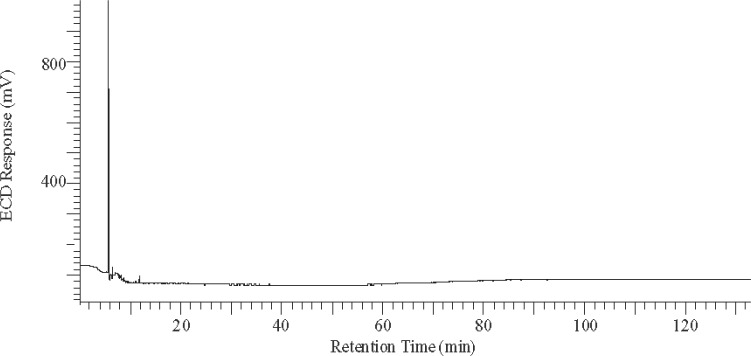
Example GC-ECD chromatogram of RM 8504, Transformer Oil, from a relatively non-polar column. Sample was first cleaned-up using solid phase extraction and normal phase liquid chromatography.

Examination of GC-ECD traces of RM 8504 samples (Fig. 1) demonstrates that PCBs, as Aroclors and as evident by electron capture detection, are not detectable in the oil. Based on the dilutions of selected Arolcor SRMs, it can be stated that Arocolor levels are < 0.1 mg/kg in RM 8504. This value corresponds to < 0.089 mg/L using the reported density of the transformer oil [1]. By comparison, non-PCB waste is classified as those materials that have PCB concentrations < 50 mg/kg by the U.S. EPA [3] and European regulations [4]. Historical (i.e., about 25 years ago) GC-ECD limits of detection reported for PCBs, as Aroclors, in transformer fluids are on the order of 0.5 mg/kg; and for PCBs as total PCBs, limits of detection range from 0.5 mg/kg to 1 mg/kg [5]. More recently, detection limits for selected Aroclors determined by liquid-liquid partitioning followed by headspace solid-phase microextraction and gas chromatography atomic emission detection are reported as 0.5 mg/L to 1 mg/L [4]. Given that the concentrations of Aroclors in the transformer oil SRMs [1] range from (17.1 ± 1.0) mg/kg (SRM 3075, Aroclor 1016 in Transformer Oil) to (4252 ± 115) mg/kg (SRM 3076, Aroclor 1242 in Transformer Oil), the reported less than value of PCBs, as Aroclors, in RM 8504 is well below the concentrations of Aroclors in transformer oil SRMs. RM 8504, used in conjunction with the individual Aroclors in transformer oil SRMs, will be useful to laboratories to underpin accurate determination of the concentrations of individual Aroclors, Aroclor combinations, or PCB mixtures in oils or similar matrices.

## Footnotes

1 Certain commercial equipment, instruments, or materials are identified in this paper to specify adequately the experimental procedure. Such identification does not imply recommendation or endorsement by NIST, nor does it imply that the materials or equipment identified are necessarily the best available for the purpose.
